# HIV treatment regimens and adherence to national guidelines in Australia: an analysis of dispensing data from the Australian pharmaceutical benefits scheme

**DOI:** 10.1186/s12889-018-6325-5

**Published:** 2019-01-03

**Authors:** Nila J. Dharan, Tomas Radovich, Samuel Che, Kathy Petoumenos, Prabhjot Juneja, Matthew Law, Robin Huang, Hamish McManus, Mark N. Polizzotto, Rebecca Guy, Peter Cronin, David A. Cooper, Richard T. Gray

**Affiliations:** 1 Kirby Institute, UNSW Sydney, Wallace Wurth Building, Sydney, NSW 2052 Australia; 2 Prospection Pty Ltd, Eveleigh, NSW Australia

**Keywords:** HIV, ART guidelines, Drug-drug interactions, Pharmaceutical benefits scheme, Comorbidities

## Abstract

**Background:**

Treatment guidelines for antiretroviral therapy (ART) have evolved to emphasize newer regimens that address ageing-related comorbidities. Using national Australian dispensing data we compare ART regimens with Australian HIV treatment guidelines in the context of treated comorbidities.

**Methods:**

The study population included all individuals in a 10% sample of national data from the Australian Pharmaceutical Benefits Scheme (PBS) who purchased a prescription of ART during 2016. We defined each patient’s most recently dispensed ART regimen and characterized them to evaluate regimen complexity and adherence to national HIV treatment guidelines. We then analyzed ART regimens in the context of other co-prescriptions purchased for defined comorbidities.

**Results:**

The 1995 patients in our sample purchased 212 different ART regimens during 2016; 1524 (76.4%) purchased one of the top ten most common regimens of which 62.3% were integrase strand transfer inhibitor-based. Among the 1786 (90%) patients that purchased the most common regimens, 83.7% purchased a regimen recommended by the guidelines for initial antiretroviral therapy and 11.4% purchased antiretrovirals that are not recommended for initial therapy; < 1% of the entire cohort purchased medications not recommended for use. While most patients purchased optimal ART regimens with low potential for significant drug interactions, regimen choices in the setting of risk factors for heart disease, renal disease and low bone mineral density appeared suboptimal.

**Conclusions:**

Australian HIV providers are prescribing ART regimens in accordance with updated treatment guidelines, but could further optimize regimens in the setting of important medical comorbidities.

**Electronic supplementary material:**

The online version of this article (10.1186/s12889-018-6325-5) contains supplementary material, which is available to authorized users.

## Background

With the introduction of combination antiretroviral therapy (ART), people with HIV have been living longer lives, experiencing fewer AIDS-related health events and developing more medical comorbidities associated with the natural aging process [1, 2]. Recently, modern ART medications have become available that are less likely to cause or exacerbate such medical comorbidities and have improved long term safety profiles and reduced potential for drug interactions [3]. As a result, recent ART treatment guidelines now emphasize using these newer ART agents to improve clinical outcomes, particularly in the setting of certain co-prescriptions and ageing-related comorbidities such as dyslipidemia, heart disease and renal insufficiency [3].

Clinical guidelines contain recommendations for clinical practice aimed at achieving optimal health outcomes [4], and have been shown to change the clinical care of patients with HIV [5–7] and other medical conditions [8, 9]. Several international cohort studies have evaluated the impact of clinical guidelines on when to start ART based on CD4 thresholds [6, 7, 10] and the choice of ART regimen [11–14], and have demonstrated that closer adherence to guideline-recommended regimens is associated with improved clinical outcomes such as increased virologic control [11, 12]. However, more recent and national data on the use of specific ART regimens are limited, particularly with respect to specifically-recommended regimens in the setting of medical comorbidities. In Australia, the use of individual antiretrovirals is published in the Annual Surveillance reports [15] but data on the composition of combination regimens has been limited to cohort studies such as the Australian HIV Observational Database (AHOD) [16] and other HIV-positive patient cohorts [10, 17]. To our knowledge only one study has looked adherence to ART treatment guidelines, but did not evaluate this in the setting of comorbidities and co-prescriptions [10].

Here we report national data on dispensed ART regimens used in Australia from the Pharmaceutical Benefits Scheme (PBS). The PBS subsidizes the costs of prescription drugs (including all HIV drugs) to all residents of Australia and eligible foreign visitors and provides its dispensing data for use in research [18]. We analysed a 10% longitudinal sample of PBS data from 2016 to evaluate the types of ART regimens people with HIV are purchasing and to assess whether they are receiving care in accordance with Australia’s current HIV treatment guidelines [19] (based on the US Department of Health and Human Services Guidelines for the use of Antiretroviral Agents in HIV-1-Infected Adults and Adolescents [3]), particularly in the context of treated comorbidities and co-prescriptions.

## Methods

### Study population

The PBS data used in this analysis was provided by Prospection [20], a company that receives a 10% sample of PBS data [18] from the Australian Department of Human Services. The sample includes data on persons whose patient identification number ends in the same specified digit (excluding Repatriation Pharmaceutical Benefits Scheme patients) [21]. It is considered to be a representative sample for the Australian population [21, 22] and has been used in several studies [23, 24]. The data includes patient level de-identified prescription claims data from 2005 to the present and is updated every quarter allowing for longitudinal follow-up of individual patients. The 10% sample records limited demographic information (year of birth, age, sex, state of prescription purchase, and year of death) and has complete prescription claim data for all PBS listed drugs with HIV indications from mid-2013 to the present. We performed an internal validation of this sample in 2015 by comparing the number of people on ART in the 10% sample with a full 100% cross-sectional dataset requested separately from the PBS and found 98% concordance. Estimates of the number of people with HIV on ART are obtained from this sample and published in Australia’s Annual Surveillance Report [25].

For this analysis, we included all individuals in the 10% sample who purchased a prescription for ART from January 1, 2016 to December 31, 2016. Age was calculated as 2016 minus the year of birth. Because tenofovir is also used as a single agent for Hepatitis B treatment, patients purchasing tenofovir alone were excluded from the analysis. Tenofovir disoproxil fumarate/emtricitabine (Truvada) was not available on the PBS for pre-exposure prophylaxis (PrEP) in 2016.

### ART regimen analysis

As some patients are dispensed individual antiretrovirals at different times, we first calculated the “prescription duration” of each dispensed antiretroviral and then tested for overlap of prescription durations to determine whether patients were dispensed individual antiretrovirals concurrently (i.e. in combination with each other) or consecutively (i.e. they changed from one combination to another). Prescription durations were defined as the time from the date the first prescription was dispensed to the date the last prescription was dispensed. We did not include the duration of the last dispensed prescription because we did not want to presume a patient took every pill in their last prescription in case they switched regimens and had leftover medication.

**Fig. 1:**
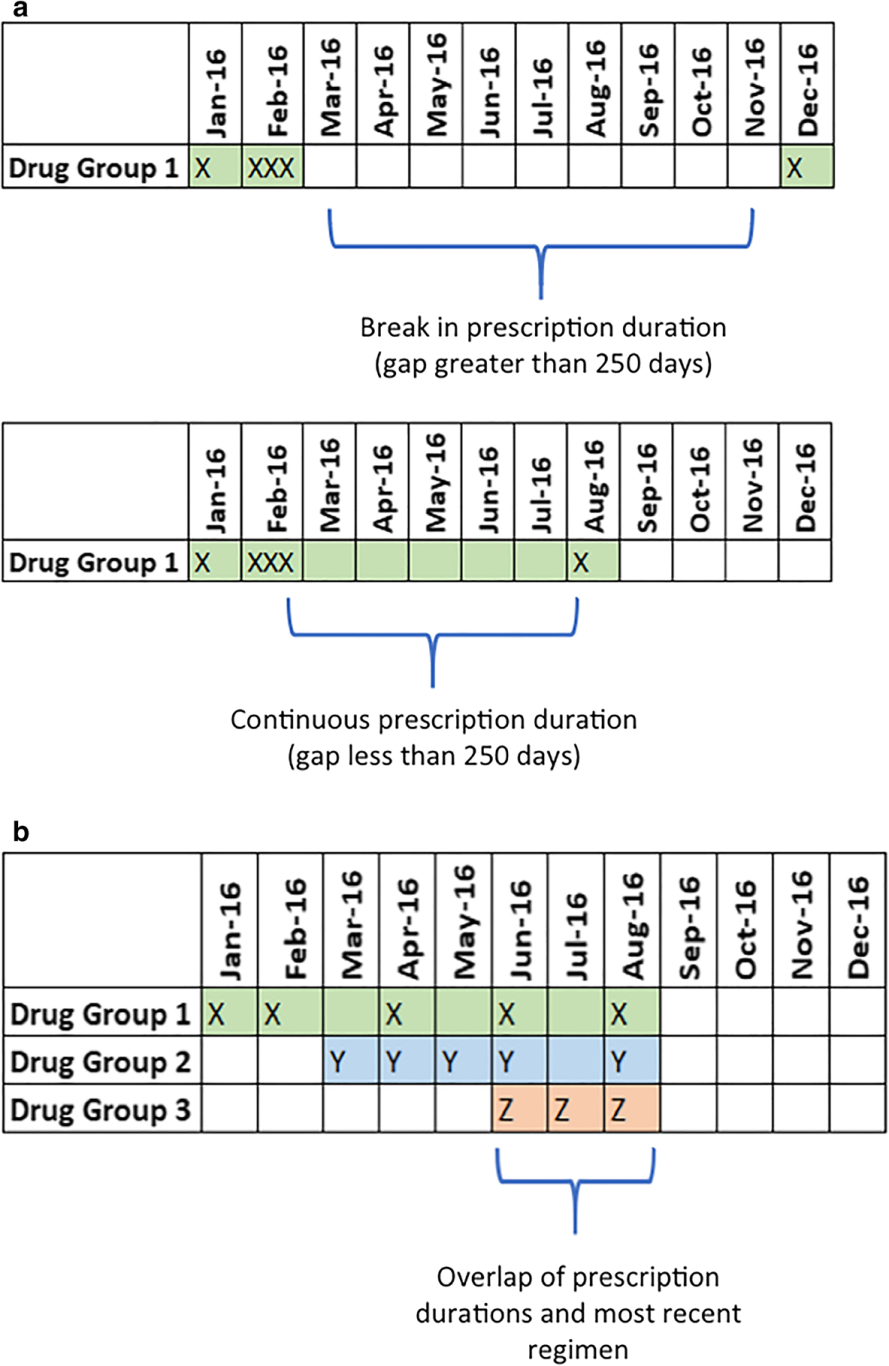
**a**. Determining the prescription duration time period. The prescription duration period for each antiretroviral was defined as the time from the date the first prescription was filled to the date the last prescription was filled. If there was any gap of 250 days or more between prescription purchases, the first prescription duration period was defined as ending on the date of the last prescription purchase, and the next prescription duration period was defined as starting on the date of the next prescription purchase. **b** Determining the patient’s most recent ART regimen. Prescription durations were determined for individual drugs and tested for overlap. The patient’s regimen was defined as the overlapping combination of drugs that was last dispensed in the 2016 calendar year

For this study we assumed that a prescription duration was on-going if there were no gaps between prescription purchases of greater than 250 days. If there was a gap greater than 250 days between consecutive prescriptions being purchased, we assumed the patient was no longer taking that antiretroviral, and the prescription duration period was determined to have ended on the date of the last prescription purchase before the gap. If there was a gap of less than 250 days we assumed the patient was potentially still taking the antiretroviral in combination with other antiretrovirals with overlapping prescription duration periods. For example, in the top panel of Fig. 1a, the first prescription duration period ended in February 2016, and the next prescription duration time period began in December 2016. We estimated the cut-off of 250 days using: the average number of days of drug per prescription pack (30 days); the PBS’s standard drop-off definition (three times the interval between purchases [26]); and the weighted average number of prescription packs filled per month (2.78): 30 days × 3 × 2.78 = 250 days. The average number of prescription packs filled per month (2.78) was estimated by taking the average number of prescription packs filled by all persons purchasing ART each month for each antiretroviral in 2016 weighted against the total number of packs of all antiretroviral drugs purchased monthly.

After the prescription duration periods for each individual antiretroviral were determined, multiple antiretroviral prescription duration periods were tested for overlap (Fig. 1b). If individual antiretrovirals overlapped the patient was considered to be on an ART regimen involving those multiple antiretrovirals. We defined the patient’s HIV regimen as the last regimen purchased during January – December 2016.

We then grouped ART regimens into six categories to characterize the types of regimens patients were being dispensed. Because retrospective PBS data were not available prior to 2013, we were unable to characterize dispensed ART regimens using each patient’s full regimen history (i.e. first line vs. second line regimens). Therefore, we developed six categories to assess, in our data from 2016, how complex ART regimens were and evaluated whether patient’s regimens at the population level were aligned with recent HIV treatment guidelines.

For the first five categories we included the top 90% of ART-purchasing patients, which included the 46 most common regimens. As the other less common regimens purchased in 2016 were purchased by very few patients (10% of the entire sample and only 20 or less patients purchasing each regimen), we felt limiting the analysis to the top 46 regimens would include data on the large majority of patients and be most representative of national ART prescribing practices. The first three categories are defined by the Australasian Society for HIV, Viral Hepatitis and Sexual Health Medicine (ASHM) Antiretroviral Guidelines on initial regimens for antiretroviral-naïve patients [27] and include: 1) “recommended” regimens (those with demonstrated virologic efficacy, high tolerability, and ease of use); 2) “alternative” regimens (those with potential disadvantages, limited use in certain patient populations, or less supporting data than recommended regimens); and 3) “other” regimens (those with decreased virologic efficacy, limited supporting data, greater toxicity or pill burden, or limited use in certain patient populations compared with “recommended” or “alternative” regimens).

Because the first three categories focus on initial regimens for antiretroviral-naïve persons, we considered three additional categories to further characterize ART treatment regimens. The fourth category included regimens containing antiretrovirals that are recommended in categories 1–3, but have been prescribed in different combinations than in the guidelines for initial therapy, such as those used in specific clinical settings such as antiretroviral resistance or end stage renal disease. A fifth category included any regimen containing antiretrovirals that would not be considered first line, but may be used in special circumstances, such as intolerance of multiple agents, and when patients have been taking the medication for a long time with good efficacy and minimal adverse effects. As noted in the ASHM guidelines, it is considered acceptable for patients to continue a regimen containing a non-recommended agent if they are virologically suppressed and tolerating the regimen well with little toxicity [27].

The sixth category included any regimen containing specific antiretrovirals that are generally not recommended and would not be prescribed by most providers. As we were evaluating rarely prescribed antiretrovirals for this category, we considered all regimens in the full dataset of 1995 patients.

### ART choices in the setting of other co-prescriptions

We next analysed ART choices in the context of other co-prescriptions, to determine whether people with HIV are being prescribed optimal ART regimens in the setting of medical comorbidities. We included patients in the co-prescriptions analysis if they had purchased ART during January–December 2016 and were alive at the end of 2016. We excluded 22 patients who died in 2016 to have 12 full months of data on co-prescriptions for each patient. We defined a co-prescription to be any drug purchased within one month before and after the purchase of an antiretroviral, as we felt it was a more accurate representation of concurrent use of both the antiretroviral and the co-prescription. For example, if a patient purchased an antiretroviral on September 15th, we considered any drug purchased from August 15th – October 15th to be a co-prescription. We performed a sensitivity analysis by expanding the time span around the antiretroviral prescription fill date to two months (in the above example, July 15th – November 15th) and three months (June 15th – December 15th) before and after the antiretroviral purchase and found minimal changes in our results.

Co-prescriptions evaluated for potential suboptimal ART choices included medication purchases with the following indications: dyslipidemia, mental illness, diabetes and bone disease (osteoporosis or low bone mineral density). While cancer is a common co-morbidity among persons with HIV, we did not analyze cancer medications here because they are not always reimbursed by the PBS in some settings. We analyzed all patients receiving medications for dyslipidemia (including both HMG Co-A reductase inhibitors, i.e. statins, such as atorvastatin, and other lipid lowering agents, such as cholestyramine and ezetimibe), to determine if they were receiving ART regimens with optimal lipid side effect profiles. We also looked at patients receiving statins and specific antiretrovirals, to see if they were being prescribed statins with the lowest potential for drug interaction. Medications with indications for diabetes treatment included all types of insulin and all types of oral hypoglycemic drugs (for example, sulfonylureas and biguanides). We restricted our analysis of osteoporosis or low bone mineral density to a population with more severe bone loss by excluding purchases of calcium and vitamin D.

Patients receiving medications for mental illness were evaluated to see whether they were receiving the non-nucleoside reverse transcriptase inhibitors (NNRTI) efavirenz or rilpivirine. Sub-categories of medications for mental illness included anti-depressants, anxiolytics, lithium for bipolar disorder and antipsychotics. The anti-depressants category included medications with indications for both depression and anxiety. Medications listed in the anti-anxiety category included benzodiazepines, which are more specifically used for anxiety alone. Similarly, medications in the antipsychotic category included those with indications for both schizophrenia and bipolar disorder, while the category of bipolar medications included lithium only.

We also evaluated ART regimen choices purchased by persons with risk factors for heart disease (those purchasing medications for diabetes, hypertension or hyperlipidemia) and persons with risk factors for renal insufficiency (those purchasing medications for diabetes or hypertension). The full list of medications included in each comorbidity category can be found in the Comorbidities Classifications spreadsheet in the Additional files 1 and 2. Lastly, specific classes of medications (proton pump inhibitors, oral contraceptives and corticosteroids) were also analysed for known drug interactions with specific antiretrovirals. The full list of medications included in the drug interactions analysis can be found in the Drug Interactions Classifications spreadsheet in the Additional files 1 and 2.

### Statistical analyses

Descriptive statistics were used to calculate the proportions of patients receiving various ART regimens and co-prescriptions in Microsoft Excel 2010.

## Results

### Patient demographics and ART regimens

Of the 1995 patients who purchased ART in our PBS sample in 2016, 88.5% were male and the median age was 49 (range 1–88) (Table 1). The largest proportion of patients came from New South Wales (39.4%), followed by Victoria (27.9%), Queensland (17.2%) and Western Australia (7.7%).

**Table 1:** Demographic characteristics of ART-purchasing persons in 2016

Characteristic	ART-purchasing cohort *N* = 1995 *n* (%)
Male gender	1765 (88.5%)
Age (median, range)^a^	49 (1–88)
≤25	52 (2.6%)
26 to 35	242 (12.1%)
36 to 45	463 (23.2%)
46 to 55	693 (34.7%)
56 to 65	374 (18.7%)
66 to 75	134 (6.7%)
76 to 85	36 (1.8%)
≥86	< 3 (< 0.15%)^b^
State of prescription purchase	
NSW	787 (39.4%)
VIC	556 (27.9%)
QLD	344 (17.2%)
WA	154 (7.7%)
SA	90 (4.5%)
TAS	24 (1.2%)
ACT	29 (1.5%)
NT	11 (0.6%)

^a^Age was calculated as the number of years from the year of birth to 2016

^b^Where results are based on less than 3 patients in the sample, a condition of data release is that the results are to be replaced with < 3

A total of 212 different ART regimens were purchased in 2016, with most patients purchasing guideline-recommended regimens. Table 2 shows the number and percentage of patients who purchased the ten most common regimens. Of the total 1995 patients included in the study, 1524 (76.4%) purchased one these 10 regimens. Of the 1524 patients that purchased one of the top ten most common regimens, 1133 (74.3%) purchased single fixed-dose combination tablets, 949 (62.3%) purchased integrase strand transfer inhibitor (InSTI)-based regimens, 534 (35.0%) purchased NNRTI-based regimens, and only 41 (2.7%) purchased protease inhibitor (PI)-based regimens (Table 2). Tenofovir disoproxil fumarate (TDF) or tenofovir alafenamide (TAF) formed the regimen backbone for 1046 patients (68.6%); of those, 256 (24.5%) had a TAF backbone. The remaining 478 (31.4%) patients that did not purchase TDF or TAF purchased regimens with an abacavir backbone.

**Table 2:** Ten most common ART regimens purchased in 2016; *n* = 1524 (76.4% of the total sample of 1995 participants)

Regimen	*N* = 1524 *n* (%)
dolutegravir/abacavir/lamivudine	419 (27.5%)
elvitegravir/cobicistat/TAF/emtricitabine	256 (16.8%)
efavirenz/TDF/emtricitabine	207 (13.6%)
rilpivirine/TDF/emtricitabine	190 (12.5%)
dolutegravir + TDF/emtricitabine	132 (8.7%)
raltegravir + TDF/emtricitabine	81 (5.3%)
nevirapine + TDF/emtricitabine	78 (5.1%)
elvitegravir/cobicistat/TDF/emtricitabine	61 (4.0%)
nevirapine + abacavir/lamivudine	59 (3.9%)
r/Atazanavir + TDF/emtricitabine	41 (2.7%)

*TDF* tenofovir disoproxil fumarate, *TAF* tenfovir alafenamide

We further characterized the 46 most common ART regimens purchased by 1786 (89.5%) of all ART-purchasing patients in 2016 (Table 3). Among these, 83.7% of patients purchased one of the three categories of guideline-recommended regimens for initial antiretroviral therapy in treatment-naïve persons [27] in 2016. A small proportion (5.0%) purchased other combinations of guideline-recommended agents (category four), and 11.4% purchased antiretrovirals that are not recommended for first-line ART but are used in special circumstances (category five). To examine category six (medications generally not recommended), we evaluated all 1995 ART-purchasing regimens in 2016 and found that only 14 (0.7%) people purchased a regimen containing a category six medication: abacavir/lamivudine/zidovudine (3), didanosine (3), stavudine (2), zidovudine (2), fosamprenavir (4), indinavir (1) (one person purchased both stavudine and indinavir), and no one purchased saquinavir. Of all category six prescriptions purchased in 2016, 63.6% of the patients purchasing these medications were over the age of 50.

**Table 3:** The 46 most common ART regimens purchased in 2016, by regimen category^a^

Regimen category^b^	*N* = 1786 *n* (%)
Category 1: Guideline recommended regimens	998 (55.9)
dolutegvravir/abacavir/lamivudine	419 (42.0)
elvitegravir/cobicistat/TAF/emtricitabine	256 (25.7)
dolutegravir + [TDF/emtricitabine or TAF/emtricitabine]	146 (14.6)
raltegravir + [TDF/emtricitabine or TAF/emtricitabine]	89 (8.9)
elvitegravir/cobicistat/TDF/emtricitabine	61 (6.1)
r/darunavir + [TDF/emtricitabine or TAF/emtricitabine]	27 (2.7)
Category 2: Guideline alternative regimens	448 (25.1)
efavirenz/TDF/emtricitabine	207 (46.2)
rilpivirine/TDF/emtricitabine	190 (42.4)
[r/atazanavir or c/atazanavir] + [TDF/emtricitabine or TAF/emtricitabine]	44 (9.8)
[c/darunavir or r/darunavir] + abacavir/lamivudine	7 (1.6)
c/darunavir + [TDF/emtricitabine or TAF/emtricitabine]	0
efavirenz + TAF/emtricitabine	0
rilpivirine/TAF/emtricitabine	0
Category 3: Guideline other regimens	48 (2.7)
raltegravir + abacavir/lamivudine	15 (31.3)
efavirenz + abacavir/lamivudine	14 (29.2)
r/darunavir + raltegravir^c^	11 (22.9)
[r/atazanavir or c/atazanavir] + abacavir/lamivudine	8 (16.7)
lopinavir/ritonavir + lamivudine^c^	0
Category 4: Other combinations of guideline-recommended ART	89 (5.0)
r/darunavir + dolutegravir	15 (16.9)
r/darunavir + raltegravir + TDF/emtricitabine	13 (14.6)
elvitegravir/cobicistat/TAF/emtricitabine + darunavir	10 (11.2)
r/lopinavir + TDF/emtricitabine	9 (10.1)
dolutegravir/abacavir/lamivudine + TDF	7 (7.8)
TDF/emtricitabine alone	6 (6.7)
elvitegravir/cobicistat/TAF/emtricitabine + atazanavir	4 (4.5)
r/darunavir + lamivudine	4 (4.5)
r/atazanavir + abacavir/lamivudine + TDF	3 (3.4)
dolutegravir/abacavir/lamivudine + r/darunavir	3 (3.4)
r/darunavir + dolutegravir + lamivudine	3 (3.4)
dolutegravir + rilpivirine + lamivudine	3 (3.4)
dolutegravir + rilpivirine	3 (3.4)
r/darunavir alone	3 (3.4)
r/darunavir + dolutegravir + TDF/emtricitabine	3 (3.4)
Category 5: Medications not recommended for first-line ART regimens but used in special circumstances	203 (11.4)^d^
nevirapine-containing regimen^d^	157
etravirine-containing regimen	20
unboosted atazanavir or darunavir-containing regimen	18
zidovudine/lamivudine-containing regimen^c^	16
maraviroc-containing regimen	4
tipranavir-containing regimen	0
enfurvitide-containing regimen	0

^a^This table excludes category six which is reported in the text

^b^Regimens were grouped together if containing the same individual antiviral agents; for example, the combination tablet dolutegravir/abacavir/lamivudine was grouped with regimens of dolutegravir plus the combination tablet abacavir/lamivudine and with regimens of the three individual medications dolutegravir plus abacavir plus lamivudine, if prescribed separately

^c^Recommended for use only when TAF, TDF or ABC cannot be used

^d^Total number of persons purchasing any antiretroviral listed in category five. Twelve people were purchasing both nevirapine and zidovudine/lamivudine; therefore, 203 persons were purchasing 215 medications in category five

### ART choices and other co-prescriptions

Of the 1995 patients who purchased ART in 2016, 22 of them died in 2016 and were excluded from the co-prescriptions analysis. While most ART-purchasing patients purchased preferred ART regimens considering their co-prescription purchases (Table 4), regimen choices for patients who purchased medications for risk factors for heart disease (hyperlipidemia, hypertension or diabetes), renal disease (hypertension or diabetes), and low bone mineral density could be improved. Among the 19.5% of patients who purchased a prescription for a hyperlipidemia, 55.6% purchased an ART regimen that contained a PI, efavirenz, or elvitegravir, which are associated with dyslipidemia [3]. However, of the 105 persons who purchased a statin and a protease inhibitor, the majority (94.3%) purchased a statin with decreased potential for drug interaction (66 purchased rosuvastatin, 33 purchased atorvastatin), and 10 (9.5%) purchased pravastatin and one purchased fluvastatin which are considered optimal agents for reducing the potential for drug interaction with PIs [3, 28]. Only five (4.7%) purchased simvastatin, which is contraindicated for most PIs. Some patients purchased more than one type of statin during the year.

**Table 4:** ART choices in the setting of common co-prescriptions

Patients who purchased medication for specified class of co-prescription^a^	*N* =1973^b^ *n* (%)
Dyslipidemia (any lipid-lowering medication)	385 (19.5)
Purchased protease inhibitors^c^	112 (29.1)
darunavir	71 (63.4)
atazanavir	36 (32.1)
r/lopinavir	9 (8.0)
fosamprenavir	1 (0.8)
Purchased efavirenz	60 (15.6)
Purchased elvitegravir	42 (10.9)
Purchased abacavir	135 (35.1)
Hypertension or hyperlipidemia or diabetes (at risk for cardiovascular disease)	574 (29.1)
Purchased any protease inhibitor^c^	155 (27.0)
darunavir	93 (60.0)
atazanavir	50 (32.3)
r/lopinavir	12 (7.7)
fosamprenavir	2 (1.3)
Purchased elvitegravir	74 (12.9)
Purchased abacavir	197 (34.3)
Hypertension or diabetes (at risk for renal disease)	413 (20.9)
Purchased TDF	230 (55.7)
Osteoporosis or low bone mineral density	27 (1.4)
Purchased tenofovir disoproxil fumarate	13 (48.1)
Mental illness	525 (26.6%)
Purchased rilpivirine	79 (15.0)
Purchased efavirenz	38 (7.2)
Diabetes	91 (4.6)
Purchased protease inhibitors^c^	33 (36.2)
Purchased ritonavir-containing regimens	24 (26.3)
Proton pump inhibitors	281 (14.2)
Purchased rilpivirine	15 (5.3)
Purchased atazanavir	11 (3.9)
Oral contraceptives	12 (0.6)
Purchased protease inhibitors^c^	4 (33.3)
Purchased efavirenz	3 (25.0)
Purchased elvitegravir	1 (8.3)
Corticosteroids (oral and inhaled except beclomethasone)	179 (9.1)
Purchased protease inhibitors^c^	46 (25.7)

^a^Co-prescriptions purchased within a month before or after the antiretroviral purchase were analysed

^b^Twenty-two patients of the 1995 patients that purchased ART in 2016 died during 2016 and were excluded from this analysis

^c^boosted with ritonavir or cobicistat or unboosted

There were 574 (29.1%) ART-purchasing patients who also purchased any medication for hypertension or hyperlipidemia or diabetes (risk factors for cardiovascular disease). Of these, 27.0% purchased a PI, 34.3% purchased abacavir, and 12.9% purchased elvitegravir. Among the 155 that purchased a PI, 7.7% purchased lopinavir/ritonavir, 60.0% purchased darunavir, and two (1.3)% purchased fosamprenavir; 32.3% purchased atazanavir which has been less associated with increased risk of cardiovascular events. Among the 413 (20.9%) of ART-purchasing patients who also purchased medication for diabetes or hypertension (risk factors for renal disease), 55.7% also purchased TDF, which has nephrotoxic side effects. A small minority (1.4%) of patients purchased prescription medications for osteoporosis or low bone mineral density, but of these, 13 (48.1%) also purchased TDF, which can lower bone mineral density.

Among the 26.6% of patients who purchased a prescription for mental illness, 22.2% purchased efavirenz or rilpivirine, which have a higher risk of central nervous system side effects. However, of those, the majority (68%) purchased rilpivirine, which is preferred over efavirenz. We also evaluated the proportion of patients who purchased various types of mental health medications (anti-depressants, benzodiazepines, lithium and antipsychotics), and found a similar trend: a minority (15–33% in each category) purchased either efavirenz or rilpivirine but the majority purchased rilpivirine (data not shown).

Only 4.6% of ART-purchasing persons also purchased a medication for diabetes. Among those, a third purchased protease inhibitors and 26.3% purchased ritonavir, which can worsen insulin resistance and impair glucose tolerance [29]. Among the 14.2% of patients who purchased proton pump inhibitors, only 9.2% purchased atazanavir and/or rilpivirine. About a third of patients who purchased oral contraceptives also purchased a PI, and 25% purchased efavirenz, which can interact with oral contraceptives. Similarly, a quarter of patients who purchased corticosteroids also purchased protease inhibitors that can inhibit the metabolism of corticosteroids.

## Discussion

Based on a nationally representative 10% sample of PBS data, a total of 1995 persons purchased ART in Australia in 2016, corresponding to a national estimate of 19,950 persons on ART. There were 212 ART regimens purchased; 76% of patients purchased one of the top 10 most common regimens and 90% purchased one of the top 46 most common regimens. Overall, 1494 (84%) of those purchasing one of the top 46 most common regimens purchased regimens that are guideline “recommended”, “alternative” or “other” for initial therapy in antiretroviral naïve patients, corresponding to around 14,940 people nationally. A minority (203; 11%) purchased medications that most HIV treatment providers would not recommend as first-line, corresponding to around 2030 people nationally. The majority (62–75%) purchased single fixed-dose combination tablets, InSTI-based regimens and tenofovir-containing regimens. About 25% of persons purchasing tenofovir-containing regimens purchased TAF, which only became available for use in Australia in April of 2016 (as part of the fixed-dose combination tablet elvitegravir/cobicistat/TAF/emtricitabine), the year of the data we analysed. While we found that most patients purchased appropriate ART in the setting of purchasing certain co-prescriptions, physician choices of ART in the setting of risk factors for cardiac disease, renal disease, and low bone mineral density may be suboptimal.

Our results are aligned with data from other international [11, 12] and Australian [10, 17, 25] cohort studies. As we found in our analysis, 84% of the ten most common ART regimens taken in 2016 by AHOD participants (the largest Australian HIV cohort) [25], were guideline “recommended”, “alternative” or “other” regimens. An older cohort study of 500 patients that compared ART regimens with guidelines also found similar proportions of participants that received drugs in the guidelines “preferred” category (69%) and the guideline “preferred” or “alternative” categories (86%) [10]. In contrast, both AHOD, whose participants have had HIV for longer than the average patient in our PBS data, and a smaller HIV cohort study [17], whose participants were enrolled before dolutegravir/abacavir/lamivudine and elvitegravir/cobicistat/TAF/emtricitabine became widely-used in Australia [25, 30], found fewer ART regimens were InSTI-based and more were PI-based. Considering our data is more recent and representative of all patients with HIV in Australia, these differences suggest that HIV prescribers are updating their ART prescribing practice in accordance with current guidelines, as has been previously reported [5, 10, 31]. In addition, of the small minority (< 1%) of all ART-purchasing patients that purchased category six antiretrovirals, the majority (64%) was over the age of 50. Given that the median age of persons with newly-diagnosed HIV in Australia is 34 [25], this suggests that most of these patients have been on these medications for over a decade.

Our data suggest that physician choices of ART may be suboptimal in patients with dyslipidemia and other risk factors for cardiovascular disease. We also found most patients purchasing both PIs and statins did not purchase statins with the lowest potential for drug interaction, as was reported in another recent cohort study [17]. With the availability of InSTI that have improved lipid profiles and reduced potential for drug interaction with statins [3], patients with dyslipidemia and other cardiac risk factors should be considered for InSTI-based therapy. Furthermore, 34% of patients who purchased medication for hypertension, dyslipidemia or diabetes also purchased abacavir, which is generally not recommended for persons at high risk for heart disease [3, 32, 33]. Similarly, 48% of patients who purchased prescriptions for osteoporosis or low bone mineral density and 56% of those who purchased prescriptions for hypertension or diabetes (risk factors for renal disease) were purchasing TDF, which can cause bone mineral density loss and nephrotoxicity.

Our results are subject to several limitations. As the 10% sample of PBS data is representative of the overall population in Australia, our results cannot necessarily be generalized to other populations with different demographic and/or socioeconomic characteristics. The PBS dataset contains limited sociodemographic and clinical data and we were unable to contextualize our findings relative to such factors, or correlate our findings of adherence to guidelines with clinical outcomes. In addition, due to the limited longitudinal retrospective data we were unable to interpret regimens in the context of prior treatment history (i.e. whether regimens were first-line vs. second-line). Some people with HIV on ART may not be captured if they did not receive PBS-funded ART in 2016, such as temporary residents ineligible for Medicare, but these numbers are very small [27]. The prescription duration period was used to determine whether medications were purchased together or separately. However, the PBS dataset only captures reimbursed prescriptions, and medications given to patients while hospitalized or through participation in clinical trials including antiretrovirals and cancer medications would be missed. This may explain why we found some patients were dispensed ritonavir/darunavir alone. In addition, medication may have been passed on to others, such as TDF/emtracitabine that is used for pre-exposure prophylaxis and was not readily available in Australia for that purpose during the study. We only included co-prescriptions purchased within 30 days before and after the ART purchase and we did not take into consideration the prescription duration. Therefore, co-prescriptions that are filled infrequently, such as those taken on an as needed basis or those that can be filled for longer than 30 days would be missed. However, expanding the co-prescription window did not yield significantly different results in our sensitivity analysis, and most common co-prescriptions are restricted to 30 days as per PBS policy [34]. Lastly, we were unable to evaluate for suboptimal ART choices in the setting of all clinical scenarios, as we relied upon a prescription marker to identify a potential comorbidity. Despite these considerations, compared with clinical data on prescribed ART regimens reported in cohort studies that were obtained from medical record review, our data provide a unique opportunity to evaluate ART regimen dispensing on a national scale, which smaller cohort studies are unable to do.

## Conclusions

While Australian providers are prescribing ART regimens in accordance with up-to-date guidelines, there is room for improvement in the management of medical comorbidities, particularly related to cardiovascular disease, renal disease and low bone mineral density. As the HIV population is ageing internationally [1, 2] and in Australia [25], and studies have shown an increased prevalence of medical comorbidities in people with HIV compared to people without HIV [35–38], prescribing optimal ART regimens in the setting of comorbidities and co-prescriptions has become a central component of clinical HIV care. Pharmacists, as well as ART prescribers, should also be aware of optimal ART regimens in the setting of specific comorbidities and complete patient co-medication dispensing data should be available to pharmacies. Ensuring HIV treatment providers are aware of optimal ART treatment regimens as they are developed is important to improve the management of ageing-related medical comorbidities, minimize potential drug interactions in the setting of polypharmacy, and improve the quality of lives of aging people with HIV in Australia.

## Additional files

Additional file 1: Kirby MSD HIV Comorbidity Analysis - PBS Code References. This spreadsheet contains the groupings of medications used to determine comorbidity classifications (XLSX 162 kb)

Additional file 2: Kirby MSD HIV Drug Interactions Analysis - PBS Code References. This spreadsheet contains the groupings of medications used to determine drug interaction classifications (XLSX 128 kb)

## Abbreviations

AHOD: Australian HIV Observational Database
AIDS: Acquire Immunodeficiency Syndrome
ART: Antiretroviral therapy
ASHM: Australasian Society for HIV, Viral Hepatitis and Sexual Health Medicine
HIV: Human Immunodeficiency virus
InSTI: Integrase strand transfer inhibitor
NNRTI: non-nucleoside reverse transcriptase inhibitor
TAF: Tenofovir alafenamide
TDF: Tenofovir disoproxil fumarate
PI: Protease inhibitor
PBS: Pharmaceutical Benefits Scheme
